# A novel fully implicit method for calculating fluid saturation distribution in immiscible gas flooding and its application

**DOI:** 10.1038/s41598-026-59057-x

**Published:** 2026-06-23

**Authors:** Weihua Dai

**Affiliations:** Research Institute, CNOOC International Limited, Beijing, China

**Keywords:** Immiscible gas flooding, Fluid saturation, Fully implicit calculation, Energy science and technology, Engineering, Hydrology, Mathematics and computing, Physics

## Abstract

Compared with water-flooding reservoirs, gas-flooding reservoirs exhibit more complex characteristics, primarily attributed to the significant viscosity difference between the displacing phase and the displaced phase. Under the influence of the equation of state, gas viscosity and deviation factor are binary functions of temperature and pressure. As the reservoir recovery degree increases, regardless of the injection strategy employed, the reservoir pressure will present a complex heterogeneous spatial distribution. When analyzing the variation of gas saturation at different positions during the production process, it is necessary to consider the effects of reservoir pressure at the corresponding location on gas viscosity and compressibility. An extensive literature review reveals that most publicly published studies on fluid saturation in gas-flooding reservoirs are based on numerical reservoir simulation methods, where the gas state equation is introduced for quantitative description. Based on the Buckley-Leverett water-flooding equation, this study proceeds as follows: ① Firstly, a one-dimensional homogeneous radial flow tube model is established. Considering that the fractional flow curve is an S-shaped nonlinear function, a novel fully implicit method is adopted to automatically search and calculate the optimal water saturation value at each spatial step and time step. ② Secondly, considering the heterogeneity of the actual model, a multi-flow tube model accounting for permeability variation is further constructed; the fingering phenomenon of the displacing phase considering heterogeneity can be obtained by averaging the saturation distribution changes of different flow tubes. ③ Thirdly, considering the compressibility and viscosity variation of gas, the volume conservation in the water-flooding mode is converted into the mass conservation in the gas-flooding mode. Subsequently, combined with the Buckley-Leverett equation and the fully implicit solution applied to the multi-flow tube model, the fluid saturation distribution of immiscible gas flooding can be derived. The proposed method in this paper is applied to interpret and analyze the field data of an actual restricted channel gas-flooding case, and the theoretically calculated gas breakthrough time of the oil well is in high agreement with the actual data. This research method features rigorous theoretical derivation and has been verified to be feasible through practical application, thereby providing certain practical guidance and reference significance for reservoir engineering researchers.

## Introduction

When the natural energy of an oilfield is insufficient, artificial methods must be adopted to supplement formation energy for development and production. Common approaches mainly include water injection, gas injection, and water-alternating-gas (WAG) injection, with the third being a relatively special case^1–3^. Artificial water flooding is a conventional and mature development method for supplementing formation energy. Gas injection development is also applied offshore or under other special circumstances, such as when a sufficient natural gas supply is available, or when associated gas from the oilfield is difficult to transport or dispose of externally. In such cases, artificial gas injection serves as a primary method to supplement formation energy for oilfield development^4,5^.

The production performance of gas injection development is more complex than that of water flooding. As the displacing phase, gas has a significant viscosity difference from the displaced phase, which easily leads to the breakthrough of the displacing phase, namely gas channeling. To control gas channeling and improve gas flooding performance, research on the areal gas saturation distribution in gas injection development units is particularly critical. Extensive literature review shows that the industry has carried out numerous studies on the areal gas saturation distribution in gas flooding reservoirs. Given the complexity of the gas equation of state (EOS), relevant studies are mainly focused on reservoir numerical simulation. Reservoir engineers establish matched geological reservoir models and injection-production models according to the development and production characteristics of different gas flooding reservoirs. They conduct sensitivity analysis on key parameters such as injection-production well pattern and gas injection rate to observe the flow behavior of the displacing phase^6,7^, so as to quantitatively analyze the interwell gas saturation distribution^8^. On this basis, they optimize the development well pattern and gas injection parameters to enhance displacement efficiency. This is a typical research method for gas flooding reservoirs. However, reservoir numerical simulation involves multiple professions and disciplines, with complicated operation and long time consumption. Therefore, it is necessary to explore a numerical method with simpler operation while maintaining accuracy, to quantitatively analyze the interwell gas saturation distribution in gas flooding reservoirs, and further predict the timing of initial gas breakthrough in oil wells and the rising law of gas-oil ratio (GOR).

Based on the fundamental theories of seepage mechanics, the author applies the Buckley-Leverett equation for water flooding to the research on fluid saturation during immiscible gas flooding^9–11^. First, a one-dimensional homogeneous radial stream tube model is established, and the fractional flow formula is derived from the relative permeability curves. Since the fractional flow curve is a strongly nonlinear function, a novel fully implicit method is adopted to automatically search for the optimal water saturation at each spatial and time step, so as to guarantee absolute computational stability and convergence. Furthermore, considering the heterogeneity of practical reservoirs, a multi-stream tube model with mixed physical properties is constructed. The fingering of the displacing phase under heterogeneous conditions can be obtained by arithmetically averaging the saturation distribution variations of individual stream tubes. The arithmetic averaging is merely a mathematical approach, which can be flexibly applied according to field conditions. For instance, multiple stream tubes with identical properties can be used instead of a single one. In addition, segmented and differentiated assembly of a single stream tube can be adopted to address the variation of physical properties between the injection end and production end. Given the particularity of the gas state Eqs^12,13^., the volume conservation principle in water flooding equations is converted into the mass conservation principle for gas flooding. Combined with the Buckley-Leverett equation, the multi-stream tube model is solved via the fully implicit scheme to obtain the fluid saturation distribution of immiscible gas flooding. As the oil recovery factor rises during field production, the properties of injected gas and formation crude oil may alter, possibly leading to a transition from immiscible to partially miscible flow. In such cases, additional dedicated models need to be developed accordingly.

Finally, interpretation and analysis are carried out in combination with actual field data from a gas flooding project in a restricted channel reservoir. The theoretically calculated gas breakthrough time of the oil well is in good agreement with the actual field data. The proposed method features rigorous theoretical derivation and feasible practical operation, and can provide practical guidance and reference value for reservoir engineering researchers.

## Numerical solution of Buckley-Leverett equation with a novel fully implicit scheme

The governing equation for water saturation distribution corresponding to the one-dimensional homogeneous flow tube model is^14^:

1 $$\:\frac{\partial\:{\mathrm{S}}_{\mathrm{w}}}{\partial\:\mathrm{t}}+\frac{\partial\:{\mathrm{f}}_{\mathrm{w}}\left({\mathrm{S}}_{\mathrm{w}}\right)}{\partial\:\mathrm{x}}=0$$

In the above equation,$\:{\mathrm{S}}_{\mathrm{w}}$ is water saturation, in decimal fractions; $\:{\mathrm{f}}_{\mathrm{w}}\left({\mathrm{S}}_{\mathrm{w}}\right)$ is water cut, with the unit of %. Among them, $\:{\mathrm{f}}_{\mathrm{w}}\left({\mathrm{S}}_{\mathrm{w}}\right)$ can be further expressed as:

2 $$\:{\mathrm{f}}_{\mathrm{w}}\left({\mathrm{S}}_{\mathrm{w}}\right)=\frac{{\mathrm{k}}_{\mathrm{r}\mathrm{w}}\left({\mathrm{S}}_{\mathrm{w}}\right)/{{\upmu\:}}_{\mathrm{w}}}{{\mathrm{k}}_{\mathrm{r}\mathrm{w}}\left({\mathrm{S}}_{\mathrm{w}}\right)/{{\upmu\:}}_{\mathrm{w}}+{\mathrm{k}}_{\mathrm{r}\mathrm{o}}\left({\mathrm{S}}_{\mathrm{w}}\right)/{{\upmu\:}}_{\mathrm{o}}}$$

3 $$\:{\mathrm{k}}_{\mathrm{r}\mathrm{w}}\left({\mathrm{S}}_{\mathrm{w}}\right)={\left(\frac{{\mathrm{S}}_{\mathrm{w}}-{\mathrm{S}}_{\mathrm{w}\mathrm{c}}}{1-{\mathrm{S}}_{\mathrm{w}\mathrm{c}}-{\mathrm{S}}_{\mathrm{o}\mathrm{r}}}\right)}^{{\mathrm{n}}_{\mathrm{w}}}$$

4 $$\:{\mathrm{k}}_{\mathrm{r}\mathrm{o}}\left({\mathrm{S}}_{\mathrm{w}}\right)={(1-\frac{{\mathrm{S}}_{\mathrm{w}}-{\mathrm{S}}_{\mathrm{w}\mathrm{c}}}{1-{\mathrm{S}}_{\mathrm{w}\mathrm{c}}-{\mathrm{S}}_{\mathrm{o}\mathrm{r}}})}^{{\mathrm{n}}_{\mathrm{o}}}$$

In Eqs. (2)–(4), $\:{\mathrm{k}}_{\mathrm{r}\mathrm{w}}\left({\mathrm{S}}_{\mathrm{w}}\right)$ and $\:{\mathrm{k}}_{\mathrm{r}\mathrm{o}}\left({\mathrm{S}}_{\mathrm{w}}\right)$ denote the relative permeabilities of the water phase and oil phase respectively^15^, dimensionless;$\:{{\upmu\:}}_{\mathrm{w}}$ and $\:{{\upmu\:}}_{\mathrm{o}}$ represent the viscosity of water and oil respectively, with the unit of mPa·s; $\:{\mathrm{n}}_{\mathrm{w}}$ and $\:{\mathrm{n}}_{\mathrm{o}}$ stand for the water phase exponent and oil phase exponent respectively^16^, dimensionless;$\:{\mathrm{S}}_{\mathrm{o}\mathrm{r}}$ and $\:{\mathrm{S}}_{\mathrm{w}\mathrm{c}}$ are the initial residual oil saturation and initial irreducible water saturation respectively, in decimal fractions.

To solve Eq. (1), definite conditions must be specified, including initial conditions and inner boundary condition.Assume the length of the one-dimensional model is L, with the left side as the injection end and the right side as the production end.It is a binary function of distance from the injection end (x) and time (t), where the initial condition is given by:

5 $$\:\left\{\begin{array}{c}{\mathrm{S}}_{\mathrm{w}}(x,0)=1-{\mathrm{S}}_{\mathrm{o}\mathrm{r}}\:\:\:\:\:\:\:\:\:\:\:\:\:\:\:(x=0)\\\:{\mathrm{S}}_{\mathrm{w}}(x,0)={\mathrm{S}}_{\mathrm{w}\mathrm{c}}\:\:\:\:\:\:\:\:\:\:\:\:\:(0<x\le\:L)\end{array}\right.$$

The inner boundary condition is given by:

6 $$\:{\mathrm{S}}_{\mathrm{w}}(0,\mathrm{t})=1-{\mathrm{S}}_{\mathrm{o}\mathrm{r}}$$

The outer boundary condition is defined based on injection-production balance, requiring no special specification.

The length L of the one-dimensional model is divided into N uniform grids, and the spatial grid step is:

7 $$\:\varDelta\:\mathrm{x}=\mathrm{L}/\mathrm{N}$$

*N* + 1 nodes are defined in the spatial dimension, denoted as $\:{\mathrm{x}}_{\mathrm{i}}=\mathrm{i}\varDelta\:\mathrm{x}$ (i=0,1,2,…,N).Let the total calculation time be T, which is divided into M equal intervals, and the time step is:

8 $$\:\varDelta\:\mathrm{t}=\mathrm{T}/\mathrm{M}$$

M + 1 nodes are defined in the time dimension, denoted as $\:{\mathrm{t}}_{\mathrm{n}}=\mathrm{n}\varDelta\:\mathrm{t}$, where *n*=0,1,2,…,M.

Based on the above analysis, the water saturation at any grid point and any time step can be expressed as:

9 $$\:{\mathrm{S}}_{\mathrm{i}}^{\mathrm{n}}={\mathrm{S}}_{\mathrm{w}}({\mathrm{x}}_{\mathrm{i}},{\mathrm{t}}_{\mathrm{n}})={\mathrm{S}}_{\mathrm{w}}(\mathrm{i}\varDelta\:\mathrm{x},\mathrm{n}\varDelta\:\mathrm{t})$$

Equation (1) is discretized and expanded using the finite difference method at point (i, n)^17^, yielding:

10 $$\:\frac{{\mathrm{S}}_{\mathrm{i}}^{\mathrm{n}+1}-{\mathrm{S}}_{\mathrm{i}}^{\mathrm{n}}}{\varDelta\:\mathrm{t}}+\frac{{\mathrm{f}}_{\mathrm{i}}^{\mathrm{n}}-{\mathrm{f}}_{\mathrm{i}-1}^{\mathrm{n}}}{\varDelta\:\mathrm{x}}=0$$

Further transformation of Eq. (10) yields:

11 $$\:{\mathrm{S}}_{\mathrm{i}}^{\mathrm{n}+1}={\mathrm{S}}_{\mathrm{i}}^{\mathrm{n}}-\frac{\varDelta\:\mathrm{t}}{\varDelta\:\mathrm{x}}({\mathrm{f}}_{\mathrm{i}}^{\mathrm{n}}-{\mathrm{f}}_{\mathrm{i}-1}^{\mathrm{n}})$$

Since the Buckley equation is nonlinear and discontinuous^18^, the calculation using Eq. (11) is highly unstable and non-convergent.Therefore, a fully implicit solution is required^19,20^, and Eq. (11) is rearranged as:

12 $$\:{\mathrm{S}}_{\mathrm{i}}^{\mathrm{n}+1}={\mathrm{S}}_{\mathrm{i}}^{\mathrm{n}}-\frac{\varDelta\:\mathrm{t}}{\varDelta\:\mathrm{x}}({\mathrm{f}}_{\mathrm{i}}^{\mathrm{n}+1}-{\mathrm{f}}_{\mathrm{i}-1}^{\mathrm{n}+1})$$

When i = 1, Eq. (12) becomes:

13 $$\:{\mathrm{S}}_{1}^{\mathrm{n}+1}={\mathrm{S}}_{1}^{\mathrm{n}}-\frac{\varDelta\:\mathrm{t}}{\varDelta\:\mathrm{x}}\left[{\mathrm{f}}_{1}^{\mathrm{n}+1}\right({\mathrm{S}}_{1}^{\mathrm{n}+1})-{\mathrm{f}}_{0}^{\mathrm{n}+1}(1-{\mathrm{S}}_{\mathrm{o}\mathrm{r}}\left)\right]$$

For i = 2,3,…,*N* − 1, Eq. (12) becomes:

14 $$\:{\mathrm{S}}_{\mathrm{i}}^{\mathrm{n}+1}={\mathrm{S}}_{\mathrm{i}}^{\mathrm{n}}-\frac{\varDelta\:\mathrm{t}}{\varDelta\:\mathrm{x}}\left[{\mathrm{f}}_{\mathrm{i}}^{\mathrm{n}+1}\right({\mathrm{S}}_{\mathrm{i}}^{\mathrm{n}+1})-{\mathrm{f}}_{\mathrm{i}-1}^{\mathrm{n}+1}({\mathrm{S}}_{\mathrm{i}-1}^{\mathrm{n}+1}\left)\right]$$

When i = N, Eq. (12) becomes:

15 $$\:{\mathrm{S}}_{\mathrm{N}}^{\mathrm{n}+1}={\mathrm{S}}_{\mathrm{N}}^{\mathrm{n}}-\frac{\varDelta\:\mathrm{t}}{\varDelta\:\mathrm{x}}\left[{\mathrm{f}}_{\mathrm{N}}^{\mathrm{n}+1}\right({\mathrm{S}}_{\mathrm{N}}^{\mathrm{n}+1})-{\mathrm{f}}_{\mathrm{N}-1}^{\mathrm{n}+1}({\mathrm{S}}_{\mathrm{N}-1}^{\mathrm{n}+1}\left)\right]$$

Equations (13)–(15) exhibit mutual nesting on their left- and right-hand sides, making it impossible to construct a system of linear algebraic equations for iterative solution of the coefficient matrix array. In this paper, a novel fully implicit automatic intelligent search method is adopted for solution. The following analysis takes the calculation of water saturation $\:{\mathrm{S}}_{\mathrm{i}}^{\mathrm{n}+1}$ at position (i, *n* + 1) as an example.

Step 1: Before solving $\:{\mathrm{S}}_{\mathrm{i}}^{\mathrm{n}+1}$, the value of $\:{\mathrm{S}}_{\mathrm{i}-1}^{\mathrm{n}+1}$ have already been computed. According to the variation law of water saturation in the one-dimensional model, $\:{\mathrm{S}}_{\mathrm{i}}^{\mathrm{n}+1}$will definitely be less than or equal to $\:{\mathrm{S}}_{\mathrm{i}-1}^{\mathrm{n}+1}$. Therefore, the median value of the first-step search factor is set as $\:0.5({\mathrm{S}}_{\mathrm{i}-1}^{\mathrm{n}+1}+1-{\mathrm{S}}_{\mathrm{o}\mathrm{r}})$, which is split into two symmetric search factors x and y, expressed respectively as:

16 $$\:\mathrm{x}=0.5({\mathrm{S}}_{\mathrm{i}-1}^{\mathrm{n}+1}+1-{\mathrm{S}}_{\mathrm{o}\mathrm{r}})-0.5\left[0.5\right({\mathrm{S}}_{\mathrm{i}-1}^{\mathrm{n}+1}+1-{\mathrm{S}}_{\mathrm{o}\mathrm{r}})-{\mathrm{S}}_{\mathrm{i}-1}^{\mathrm{n}+1}]$$

17 $$\:\mathrm{y}=0.5({\mathrm{S}}_{\mathrm{i}-1}^{\mathrm{n}+1}+1-{\mathrm{S}}_{\mathrm{o}\mathrm{r}})+0.5[1-{\mathrm{S}}_{\mathrm{o}\mathrm{r}}-0.5({\mathrm{S}}_{\mathrm{i}-1}^{\mathrm{n}+1}+1-{\mathrm{S}}_{\mathrm{o}\mathrm{r}}\left)\right]$$

Simplifying the above expressions for x and y yields:

18 $$\:x = 0.25(1 - S_{{or}} ) + 0.75S_{{i - 1}}^{{n + 1}}$$

19 $$\:y = 0.75(1 - S_{{or}} ) - 0.25S_{{i - 1}}^{{n + 1}} )$$

Substitute x and y into Eq. (14) respectively, then compare the magnitudes of the resulting expression $\:|{\mathrm{S}}_{\mathrm{i}}^{\mathrm{n}}-\frac{\varDelta\:\mathrm{t}}{\varDelta\:\mathrm{x}}[{\mathrm{f}}_{\mathrm{i}}^{\mathrm{n}+1}\left(\mathrm{x}\right)-{\mathrm{f}}_{\mathrm{i}-1}^{\mathrm{n}+1}\left({\mathrm{S}}_{\mathrm{i}-1}^{\mathrm{n}+1}\right)]-\mathrm{x}|$ and $\:|{\mathrm{S}}_{\mathrm{i}}^{\mathrm{n}}-\frac{\varDelta\:\mathrm{t}}{\varDelta\:\mathrm{x}}[{\mathrm{f}}_{\mathrm{i}}^{\mathrm{n}+1}\left(\mathrm{y}\right)-{\mathrm{f}}_{\mathrm{i}-1}^{\mathrm{n}+1}\left({\mathrm{S}}_{\mathrm{i}-1}^{\mathrm{n}+1}\right)]-\mathrm{y}|$. If the former is smaller, x is superior and will be used as the median search factor for the next fully implicit iteration; otherwise, y will be taken as the median factor.

Step 2: If x is superior, split x into two new symmetric search factors x1 and y1, expressed respectively as:

20 $$\:\mathrm{x}1=\mathrm{x}-0.5[\mathrm{x}-{\mathrm{S}}_{\mathrm{i}-1}^{\mathrm{n}+1}]$$

21 $$\:\mathrm{y}1=\mathrm{x}+0.5[\mathrm{x}-{\mathrm{S}}_{\mathrm{i}-1}^{\mathrm{n}+1}]$$

If y is superior, split y into two new symmetric search factors x1 and y1, expressed respectively as:

22 $$\:\mathrm{x}1=\mathrm{y}-0.5[1-{\mathrm{S}}_{\mathrm{o}\mathrm{r}}-\mathrm{y}]$$

23 $$\:\mathrm{y}1=\mathrm{y}+0.5[1-{\mathrm{S}}_{\mathrm{o}\mathrm{r}}-\mathrm{y}]$$

Substitute x1 and y1 into Eq. (14) respectively, then compare the magnitudes of the resulting expression $\:|{\mathrm{S}}_{\mathrm{i}}^{\mathrm{n}}-\frac{\varDelta\:\mathrm{t}}{\varDelta\:\mathrm{x}}[{\mathrm{f}}_{\mathrm{i}}^{\mathrm{n}+1}\left(\mathrm{x}1\right)-{\mathrm{f}}_{\mathrm{i}-1}^{\mathrm{n}+1}\left({\mathrm{S}}_{\mathrm{i}-1}^{\mathrm{n}+1}\right)]-\mathrm{x}1|$ and $\:|{\mathrm{S}}_{\mathrm{i}}^{\mathrm{n}}-\frac{\varDelta\:\mathrm{t}}{\varDelta\:\mathrm{x}}[{\mathrm{f}}_{\mathrm{i}}^{\mathrm{n}+1}\left(\mathrm{y}1\right)-{\mathrm{f}}_{\mathrm{i}-1}^{\mathrm{n}+1}\left({\mathrm{S}}_{\mathrm{i}-1}^{\mathrm{n}+1}\right)]-\mathrm{y}1|$. If the former is smaller, x1 is superior and will be used as the median search factor for the next fully implicit iteration; otherwise, y1 will be taken as the median factor.

Step 3: If x1 is superior, split x1 into two new symmetric search factors x2 and y2, expressed respectively as:

24 $$\:\mathrm{x}2=\mathrm{x}1-0.5[\mathrm{x}1-{\mathrm{S}}_{\mathrm{i}-1}^{\mathrm{n}+1}]$$

25 $$\:\mathrm{y}2=\mathrm{x}1+0.5[\mathrm{x}1-{\mathrm{S}}_{\mathrm{i}-1}^{\mathrm{n}+1}]$$

If y1 is superior, split y1 into two new symmetric search factors x2 and y2, expressed respectively as:

26 $$\:\mathrm{x}2=\mathrm{y}1-0.5\left[0.5\right({\mathrm{S}}_{\mathrm{i}-1}^{\mathrm{n}+1}+1-{\mathrm{S}}_{\mathrm{o}\mathrm{r}})-\mathrm{y}1]$$

27 $$\:\mathrm{y}2=\mathrm{y}1+0.5\left[0.5\right({\mathrm{S}}_{\mathrm{i}-1}^{\mathrm{n}+1}+1-{\mathrm{S}}_{\mathrm{o}\mathrm{r}})-\mathrm{y}1]$$

Substitute x2 and y2 into Eq. (14) respectively, then compare the magnitudes of the resulting expression $\:|{\mathrm{S}}_{\mathrm{i}}^{\mathrm{n}}-\frac{\varDelta\:\mathrm{t}}{\varDelta\:\mathrm{x}}[{\mathrm{f}}_{\mathrm{i}}^{\mathrm{n}+1}\left(\mathrm{x}2\right)-{\mathrm{f}}_{\mathrm{i}-1}^{\mathrm{n}+1}\left({\mathrm{S}}_{\mathrm{i}-1}^{\mathrm{n}+1}\right)]-\mathrm{x}2|$ and $\:|{\mathrm{S}}_{\mathrm{i}}^{\mathrm{n}}-\frac{\varDelta\:\mathrm{t}}{\varDelta\:\mathrm{x}}[{\mathrm{f}}_{\mathrm{i}}^{\mathrm{n}+1}\left(\mathrm{y}2\right)-{\mathrm{f}}_{\mathrm{i}-1}^{\mathrm{n}+1}\left({\mathrm{S}}_{\mathrm{i}-1}^{\mathrm{n}+1}\right)]-\mathrm{y}2|$. If the former is smaller, x2 is superior and will be used as the median search factor for the next fully implicit iteration; otherwise, y2 will be taken as the median factor.

The above procedures are repeated iteratively. With computer programs, the fully implicit automatic intelligent search calculation can be easily performed over 100 times. In practical implementation, the convergence criterion is set such that the factor error between two successive searches is less than $\:{10}^{-5}$, which yields numerically stable and convergent results of $\:{\mathrm{S}}_{\mathrm{i}}^{\mathrm{n}+1}$. Fluid saturation values calculated at any time and spatial position using this method unconditionally satisfy stability and convergence requirements.

**Fig. 1:**
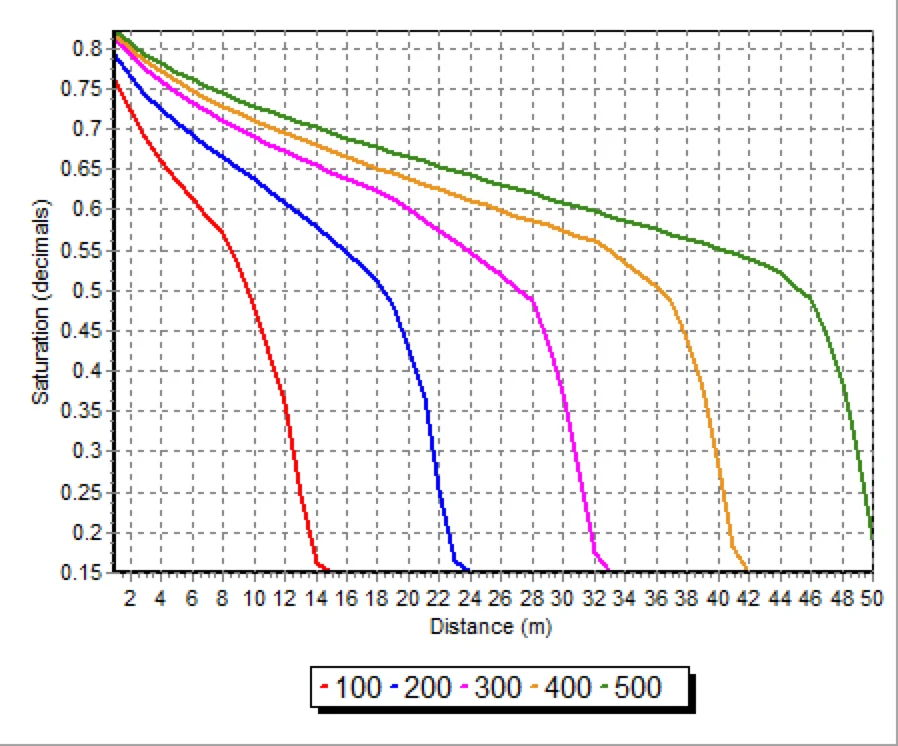
Water saturation profiles at different positions of the one-dimensional model when Swc and Sor are 0.25.

**Fig. 2:**
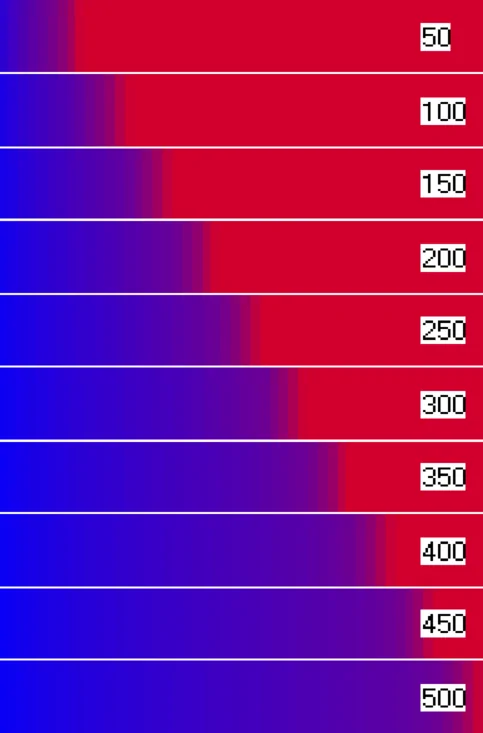
One-dimensional model water saturation profiles at different times when Swc and Sor are 0.25.

**Fig. 3:**
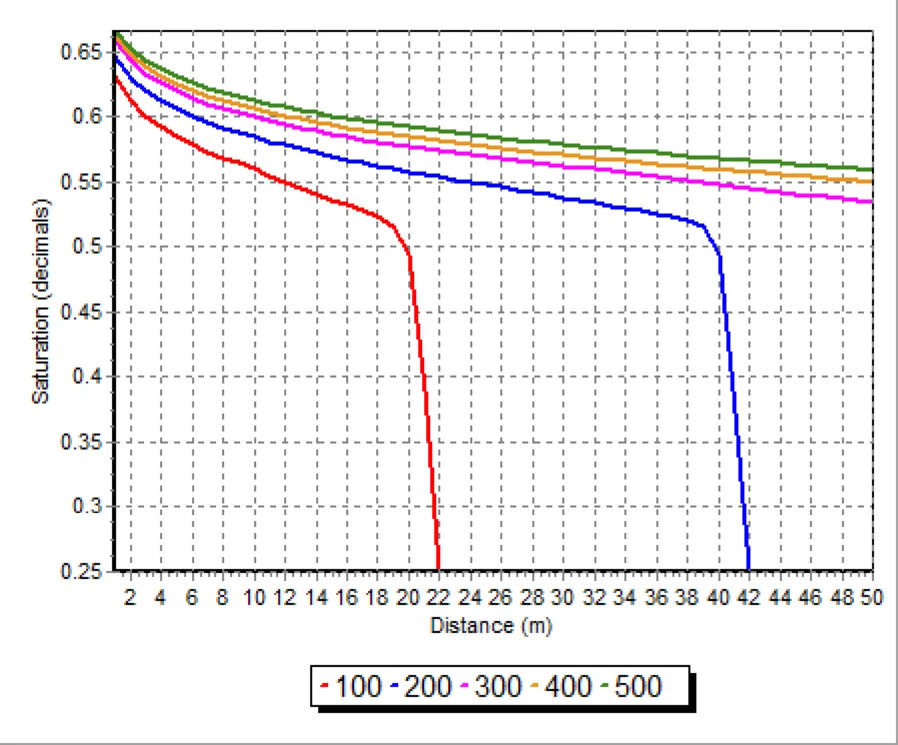
Water saturation profiles at different positions of the one-dimensional model when Swc and Sor are 0.2.

**Fig. 4:**
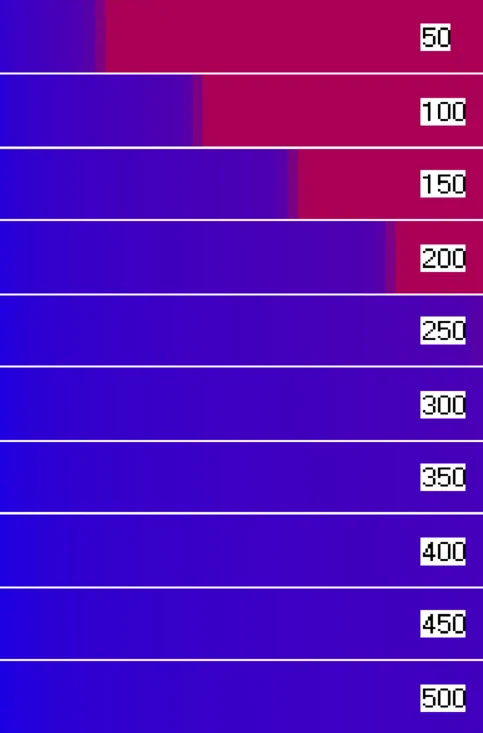
One-dimensional model water saturation profiles at different times when Swc and Sor are 0.2.

**Fig. 5:**
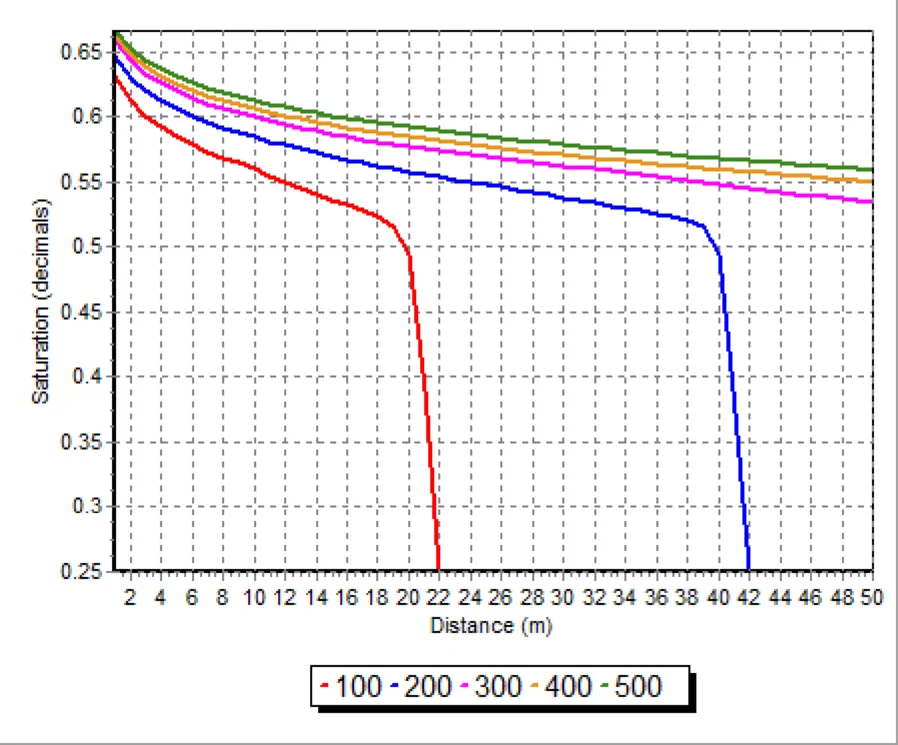
Water saturation profiles at different positions of the one-dimensional model when Swc and Sor are 0.15.

**Fig. 6:**
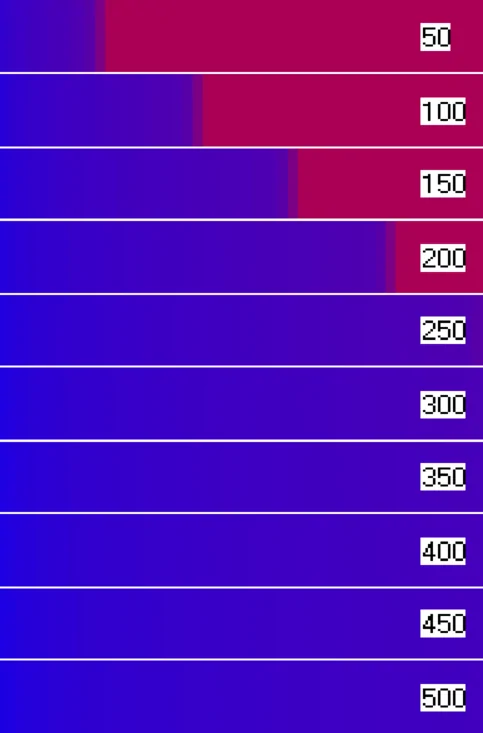
One-dimensional model water saturation profiles at different times when Swc and Sor are 0.15.

Figures 1 and 2 show water saturation profiles corresponding to different time steps, with the initial irreducible water saturation and residual oil saturation both set to 0.25. Figures 3 and 4 correspond to an irreducible water saturation and residual oil saturation of 0.2. Figures 5 and 6 correspond to an irreducible water saturation and residual oil saturation of 0.15.

In reservoir numerical simulation, the Newton-Raphson iteration method serves as the mainstream algorithm for solving nonlinear systems of equations, which is applicable to both fully implicit and semi-implicit schemes. This method takes a set of unknowns in the form of multivariate vectors, mainly including pressure and saturation. It introduces the residual vector and performs the first-order Taylor expansion. Each iteration requires solving a large sparse matrix system of equations, which accounts for the majority of computational consumption. By contrast, the fully implicit method proposed in this study is developed for one-dimensional multi-stream tube models. To avoid computational divergence, the saturation is strictly matched with pressure at each time step. For the one-dimensional stream tube model, the variation trend of saturation distribution after each time step is definite: the oil saturation gradually increases from the inlet to the outlet of the stream tube. Based on this rule, an approach similar to the bisection method is adopted to continuously search for the optimal solution, eliminating the need to solve large sparse matrix systems. This constitutes the core difference between the proposed method and the Newton-Raphson algorithm. Furthermore, adjusting the time and spatial step sizes exerts no adverse impact on computational stability and convergence. From the perspective of engineering data accuracy, the time and spatial step sizes can be appropriately reduced to improve calculation efficiency and precision when using this method.

## Multi-tube model considering reservoir heterogeneity

The one-dimensional streamtube model introduced in the second section above is equivalent to a homogeneous model. As we all know, almost all actual oil reservoirs are heterogeneous, i.e., the petrophysical properties (porosity and permeability) vary at different locations throughout the reservoir space. Therefore, a single streamtube model cannot characterize the heterogeneity of actual reservoir models. To achieve heterogeneity simulation, multiple streamtube models can be introduced^21,22^, along with multiple sets of relative permeability curves with different characteristics. The differences in these curves can be based on the degree of heterogeneity in the actual reservoir; the stronger the heterogeneity, the greater the differences in the relative permeability curves, and vice versa.

**Table 1:** Parameter characteristics of relative permeability curves in different reservoirs.

Parameters	k = 25 mD	k = 100 mD	k = 300 mD
Swc	23%–42%	23%–35%	21%–29%
Sor	22%–41%	19%–30%	14%–27%
no	3–6	2–4	1–3
nw	2–5	1.5–3	1–2
kro.max	0.55–0.70	0.70–0.85	0.80–1.00
krw.max	0.25–0.45	0.30–0.55	0.40–0.75
range of two phase	20%–50%	45%–60%	55%–75%
curve character	steep	medium	gentle

**Fig. 7:**
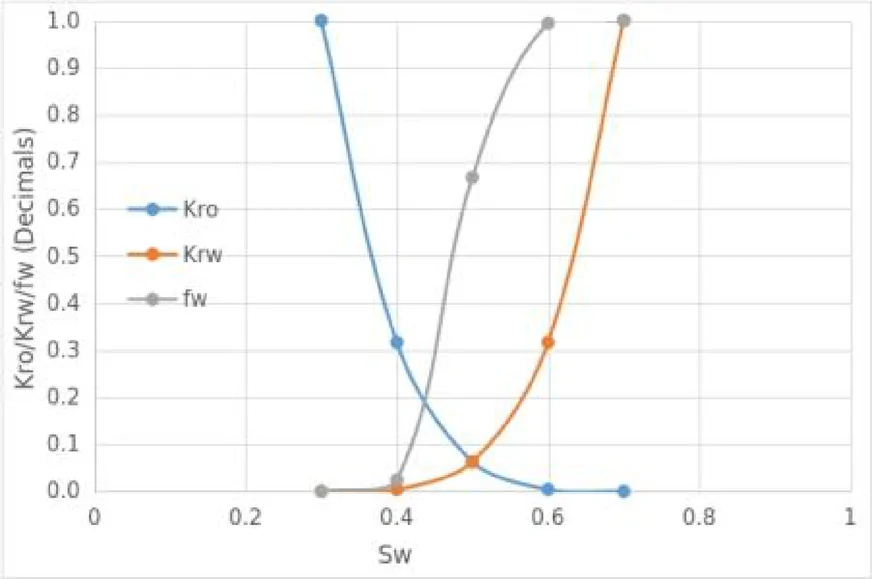
Typical relative permeability curves when permeability is 25mD.

**Fig. 8:**
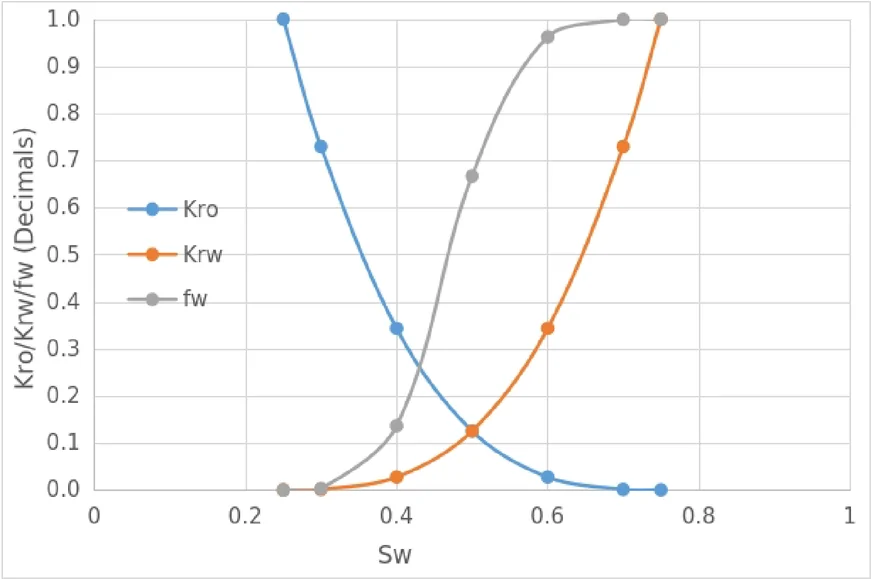
Typical relative permeability curves when permeability is 100mD.

**Fig. 9:**
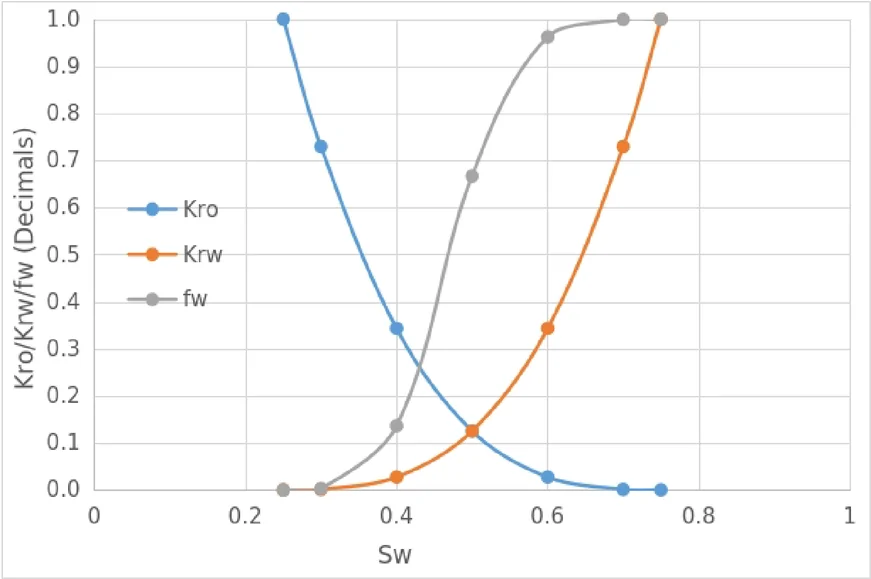
Typical relative permeability curves when permeability is 300mD.

According to the definition of relative permeability curves given by Eqs. (2–4), different petrophysical characteristics can be represented by adjusting the four parameters $S_{{wc}} {}S_{{or}} {}n_{w} {}n_{o}$. Based on the investigation and summary of actual development data from Bohai Oilfield, Table 1 lists the value ranges of the relative permeability curve parameters corresponding to permeabilities of 25 mD, 100 mD, and 300 mD, respectively.It can be seen from Table 1 that as permeability increases, the values of the four parameters decrease, the two-phase saturation region becomes wider, and the oil-phase and water-phase relative permeability curves become gentler.In low-permeability reservoirs, there are many micropores and strong capillary forces, making water displacement difficult and oil recovery inefficient. In high-permeability reservoirs, pores are dominated by large pores with uniform pore throats and weak capillary forces, resulting in lower irreducible water saturation and higher displacement efficiency. Figures 7, 8 and 9 show schematic diagrams of typical relative permeability curves corresponding to the three types of reservoirs with different petrophysical properties listed in Table 1.

For illustration: Based on the study of the one-dimensional homogeneous streamtube model, heterogeneity is considered by increasing the model to ten streamtubes with different petrophysical properties. These ten streamtubes correspond to ten sets of different relative permeability curves, and the parameters of these curves are listed in Table 2.

**Table 2:** Parameter characteristics of relative permeability curves in different reservoirs.

Parameters	No.1	No.2	No.3	No.4	No.5	No.6	No.7	No.8	No.9	No.10
Swc	0.25	0.24	0.23	0.22	0.21	0.20	0.21	0.22	0.23	0.24
Sor	0.24	0.23	0.22	0.21	0.20	0.19	0.20	0.21	0.22	0.23
no	3	3	3	3	2	2	2	3	3	3
nw	3	3	3	3	2	2	2	3	3	3
kro.max	1	1	1	1	1	1	1	1	1	1
krw.max	0.6	0.6	0.6	0.6	0.7	0.7	0.7	0.6	0.6	0.6

**Fig. 10:**
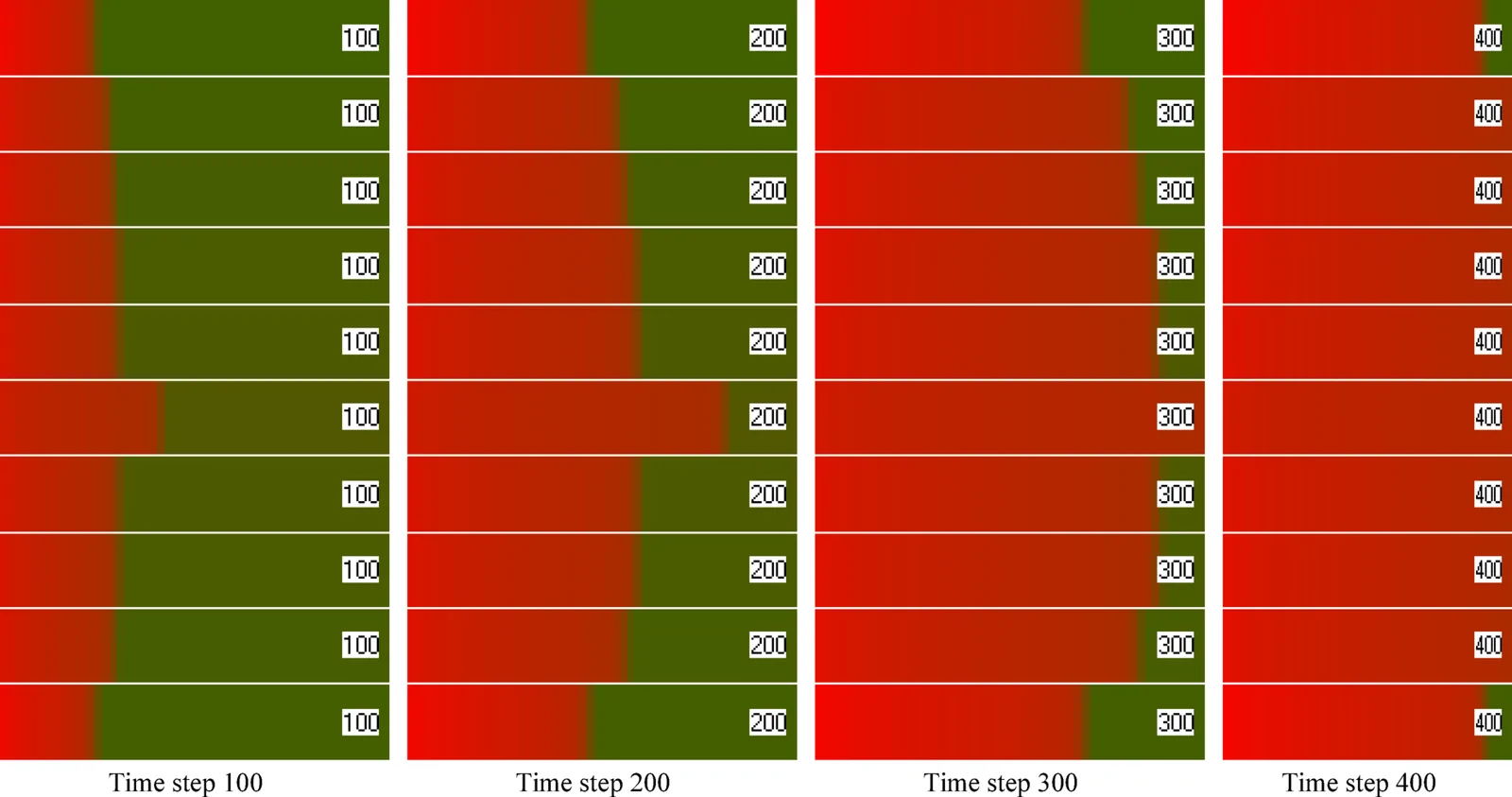
Fluid saturation profile of Diagrams of water flooding at different time steps 100, 200, 300 and 400.

Based on the data in Table 2, the governing equation for waterflooding is solved numerically using the fully implicit finite difference method for each streamtube with corresponding parameters. Figure 10 shows the waterflooding saturation profiles at different time steps of 100, 200, 300, and 400, respectively.

It can be seen from Fig. 7 that, for streamtube waterflooding models with different petrophysical properties, the waterflooding front saturation moves faster in streamtubes with higher permeability and gentler relative permeability curves. Conversely, the front advances more slowly in streamtubes with lower permeability and steeper relative permeability curves.In actual oilfield development, when high-permeability channels exist in the reservoir, injected water preferentially flows along these paths due to the tendency of fluid to move in the direction of least resistance, resulting in water breakthrough, i.e., fingering.Reservoir heterogeneity is an inherent property, so the fingering phenomenon of the displacing phase is unavoidable. However, in practical reservoir development, accurate identification of vertical high-permeability zones allows early implementation of staged completion and intelligent switching of corresponding high-permeability intervals using downhole tools. This minimizes the permeability contrast among producing zones at the same time, thereby improving the waterflood sweep efficiency and overall development performance.

The multi-stream tube flow model is applicable when the reservoir between injection wells and production wells presents a consistent directional distribution, with well-defined sedimentary patterns. In such cases, the planar distribution of sand bodies conforms to sedimentary constraints and the formation has high fluid flow capacity. Nevertheless, the model suffers from reduced simulation accuracy under strong reservoir heterogeneity, poor physical properties or high viscosity of formation crude oil. Typical scenarios include ultra-low permeability reservoirs with permeability below 10 mD, or reservoirs where the viscosity of formation crude oil exceeds 500 mPa·s. Under these circumstances, capillary and viscous effects exert non-negligible impacts on fluid flow in the reservoir.

## Application of immiscible gas flooding in Buckley-Leverett equation

When studying the fluid saturation profile during gas flooding in reservoir development, the instability and compressibility of gas must be considered. That is, with changes in pressure, gas density, compressibility, formation volume factor, viscosity, and deviation factor all vary nonlinearly. Based on Boyle-Mariotte’s law, and by converting gas volume changes into mass changes^23^, the following can be obtained:

28 $$\:{\uprho\:}=\frac{\mathrm{M}}{\mathrm{Z}\mathrm{R}\mathrm{T}}\times\:\mathrm{P}$$

The parameters in Eq. (28) are defined as follows:$\:{\uprho\:}$ is gas density, with unit$\:\mathrm{g}/{\mathrm{c}\mathrm{m}}^{3}$; M is the molar mass of gas; Z is the gas deviation factor (dimensionless), which can be calculated using the Dranchuk-Abou-Kassem method; R is the universal gas constant, 0.008315 $\:\mathrm{M}\mathrm{P}\mathrm{a}\bullet\:{\mathrm{m}}^{3}/\mathrm{k}\mathrm{m}\mathrm{o}\mathrm{l}\bullet\:\mathrm{K}$; T is gas temperature, with unit K.

The fractional flow equation for gas flooding can be expressed as:

29 $$\:f_{g} (S_{g} ,P) = \frac{{k_{{rg}} \left( {S_{g} } \right)/[\mu \:_{g} (P) \cdot B_{g} (P)]}}{{k_{{rg}} \left( {S_{g} } \right)/[\mu \:_{g} (P) \cdot B_{g} (P)] + k_{{ro}} \left( {S_{g} } \right)/\left( {\mu \:_{o} \cdot B_{o} } \right)}}$$

In Eq. (29):$\:{\mathrm{k}}_{\mathrm{r}\mathrm{g}}\left({\mathrm{S}}_{\mathrm{g}}\right)$ denotes the relative permeability of the gas phase, dimensionless;$\:{{\upmu\:}}_{\mathrm{g}}\left(\mathrm{P}\right)$ denotes the gas viscosity, a univariate function of pressure, in mPa.s, which can be calculated by the Lee-Gonzalez-Eaki theoretical formula;$\:{\mathrm{B}}_{\mathrm{g}}\left(\mathrm{P}\right)$denotes the gas formation volume factor, dimensionless, which is also a univariate function of pressure.

Based on Eq. (1) and Darcy’s law, and taking into account the variation of gas properties, the governing equations for the oil phase and gas phase can be derived separately as follows:

30 $$\:{\upvarphi\:}{{\uprho\:}}_{\mathrm{o}}\frac{\partial\:{\mathrm{S}}_{\mathrm{o}}}{\partial\:\mathrm{t}}+{{\uprho\:}}_{\mathrm{o}}\frac{\partial\:{\mathrm{v}}_{\mathrm{o}}}{\partial\:\mathrm{x}}=0$$

31 $$\:{\upvarphi\:}\frac{\partial\:\left({{\uprho\:}}_{\mathrm{g}}{\mathrm{S}}_{\mathrm{g}}\right)}{\partial\:\mathrm{t}}+\frac{\partial\:\left({{{\uprho\:}}_{\mathrm{g}}\mathrm{v}}_{\mathrm{g}}\right)}{\partial\:\mathrm{x}}=0$$

In Eqs. (30) and (31),$\:{{\uprho\:}}_{\mathrm{o}}$ is the oil density, which is generally a constant; $\:{{\uprho\:}}_{\mathrm{g}}$ is the gas density, a variable dependent on pressure, with the unit $\:\mathrm{g}/{\mathrm{c}\mathrm{m}}^{3}$;$\:{\mathrm{v}}_{\mathrm{o}}$ and $\:{\mathrm{v}}_{\mathrm{g}}$ are the flow velocities of the oil phase and gas phase, respectively, in m/d.Since there are only oil and gas phases in the reservoir, the following relation holds $\:{{\mathrm{S}}_{\mathrm{g}}+\mathrm{S}}_{\mathrm{o}}=1$. Taking the time derivative on both sides yields:

32 $$\:\frac{\partial\:{\mathrm{S}}_{\mathrm{g}}}{\partial\:\mathrm{t}}=-\frac{\partial\:{\mathrm{S}}_{\mathrm{o}}}{\partial\:\mathrm{t}}$$

Multiply both the numerator and denominator of the partial derivative of Eq. (31) with respect to x by the same quantity, and the expression can be rearranged as follows:

33 $$\:{\upvarphi\:}\frac{\partial\:}{\partial\:\mathrm{t}}\left({{\uprho\:}}_{\mathrm{g}}{\mathrm{S}}_{\mathrm{g}}\right)+\frac{\partial\:}{\partial\:\mathrm{x}}\left[{{\uprho\:}}_{\mathrm{g}}\right({\mathrm{v}}_{\mathrm{g}}+{\mathrm{v}}_{\mathrm{o}}\left)\frac{{{\uprho\:}}_{\mathrm{g}}{\mathrm{v}}_{\mathrm{g}}}{{{\uprho\:}}_{\mathrm{g}}{\mathrm{v}}_{\mathrm{g}}+{{\uprho\:}}_{\mathrm{o}}{\mathrm{v}}_{\mathrm{o}}}\right]=0$$

Here, the expressions for total fluid velocity and gas mass fraction are introduced as follows:

34 $$v = v_{g} + v_{o}$$

35 $$\:{\mathrm{F}}_{\mathrm{g}}=\frac{{{\uprho\:}}_{\mathrm{g}}{\mathrm{v}}_{\mathrm{g}}}{{{\uprho\:}}_{\mathrm{g}}{\mathrm{v}}_{\mathrm{g}}+{{\uprho\:}}_{\mathrm{o}}{\mathrm{v}}_{\mathrm{o}}}$$

Simplifying Eq. (33) and substituting Eq. (28) yields:

36 $$\:{\upvarphi\:}\frac{\partial\:}{\partial\:\mathrm{t}}\left(\frac{\mathrm{M}}{\mathrm{Z}\mathrm{R}\mathrm{T}}\mathrm{P}{\mathrm{S}}_{\mathrm{g}}\right)+\frac{\partial\:}{\partial\:\mathrm{x}}\left(\frac{\mathrm{M}}{\mathrm{Z}\mathrm{R}\mathrm{T}}\mathrm{P}\mathrm{v}{\mathrm{F}}_{\mathrm{g}}\right)=0$$

Equation (36) is the governing equation for gas saturation variation that accounts for changes in gas density and deviation factor with pressure.With reference to the theoretical derivation and computational analysis in Parts Ⅱ and Ⅲ of this paper, a fully implicit finite-difference numerical solution can be implemented for the multi-streamtube model of gas flooding. The calculated gas saturation unconditionally satisfies computational stability and convergence.In practical calculations, two typical methods are commonly used.The first method assumes negligible pressure variations between the injection section and the outlet, so the pressure can be treated as uniform across the entire streamtube. In this way, the gas phase can be approximated as a conventional fluid with constant compressibility, viscosity, and deviation factor, which allows observation of the effect of gas viscosity on displacement performance.The second method considers pressure variations between the injection and outlet ends, which can be readily obtained from experiments. The pressure profile is then discretized uniformly along the streamtube length. In actual computation, the viscosity and deviation factor corresponding to different pressures are assigned at different spatial locations, thereby completing the fully implicit finite-difference calculation of fluid saturation. None of the aforementioned theories account for the effect of gravity segregation. In practical scenarios, gravitational effects need to be considered when the vertical reservoir thickness is significant (e.g., exceeding 50 m). For this reason, appropriate simplifications have been made in this study.

Taking the ten-streamtube model as an example, the parameters for water flooding are replaced with those for gas flooding. Figure 1111 shows the dynamic variation profiles of gas-oil fluid saturation at different time steps of 20, 40, 60, and 80, respectively. It can be seen from Fig. 8 that the gas-phase flow velocity in gas flooding is significantly higher than the water-phase flow velocity in water flooding.On the one hand, this is due to the low viscosity of gas, and the relative permeability of gas is obviously higher than that of the oil phase.On the other hand, the pressure near the injection end is higher than that near the production end, which enables effective displacement. Meanwhile, the gas can rapidly expand and flow toward the direction of pressure relief, which intensifies the gas channeling phenomenon. During the calculation process of this model, the total computation time is only 28 s, and the average number of iterations required for each time step to meet the convergence accuracy requirement is 36.

## Case study

Field A is a deep water Early Cretaceous sandstone oilfield with a water depth of approximately 1800 m and a reservoir depth of about 3500 m. It features a large oil bearing area and considerable reserve volume.No faults are developed within the field; it exhibits a monoclinic structure that is high in the south and low in the north, with a gentle dip angle of about 1.5°. The reservoir is classified as a structural lithologic edge water reservoir. Laterally and vertically, the field consists of multiple superimposed single or composite sand bodies of varying scales formed in multiple stages. The reservoirs are weakly confined or unconfined turbidite channels, with an average porosity of about 23%. Permeability varies widely: core analysis shows a minimum permeability of 22mD and a maximum of up to 7000mD, classifying the reservoir as medium to high porosity and permeability. The reservoir has a normal temperature and pressure system, and the insitu crude oil is of good quality. The average formation oil viscosity is 2 mPa·s, the formation volume factor of oil is 1.4, and the average gas oil ratio is 250$\:{\mathrm{m}}^{3}/{\mathrm{m}}^{3}$.To accelerate the development of the deep water oilfield and improve development performance, full horizontal well water and gas injection are adopted. Associated gas produced is fully reinjected except for self consumption.

Sandbody C is a confined channel deposited in a single period. Its areal distribution is relatively limited, appearing approximately as an elongated belt, accounting for a small proportion of the total oilfield reserves.The development of Sandbody C employs a pattern of four horizontal wells for gas injection, consisting of two gas injectors and two oil producers, as shown in Fig. 9. Wells C1H and C4H are gas injection wells, while C2H and C3H are production wells.To evaluate the performance of gas injection development, two development wells (C1H and C2H) were implemented in the early stage. These two horizontal wells established an injector producer pair to jointly develop the reserves on the left side of Sandbody C1.

To ensure accurate evaluation of injection-production performance, wells C1H and C2H were put into production almost simultaneously. The average daily oil production of well C2H is approximately 18 kbbl, and the average daily gas injection rate of well C1H is about 100 mmscf. Due to the limited scale of the sand body, the gas-oil ratio (GOR) of well C2H began to rise only 5 months after commissioning. It remains unclear whether the variation in wellhead GOR of well C2H is a normal production characteristic or indicates local gas channeling through high-permeability streaks. Given that this reservoir model follows a narrow channel pattern, the displacement mechanism in narrow channels is similar to that of the multi-stream tube model. Changes in well configurations act as source or sink terms relative to the entire channel, and the influence of planar radial flow is relatively minor. Therefore, the research method proposed in this paper is adopted to conduct the simulation analysis.

First, the reservoir heterogeneity is analyzed based on the variations in reservoir physical properties along the horizontal sections penetrated by Wells C1H and C2H.The horizontal lengths of C1H and C2H are approximately 800 m, with permeability ranging from a minimum of 30 mD to a maximum of 1500 mD; most intervals exhibit permeability between 100 mD and 800 mD.Careful observation reveals that both horizontal wells intersect a zone with exceptionally high porosity and permeability, where permeability reaches 1000–1200 mD.Comparative analysis of the two wells indicates this is a continuously developed high permeability streak.The overall permeability contrast of the reservoir is as high as 50, showing strong heterogeneity in reservoir properties.Based on this study, a ten streamtube model with distinct physical parameters is established, and the relative permeability curve parameters are assigned individually for each streamtube.

Second, the pressure profile between Wells C1H and C2H is analyzed based on the bottomhole injection pressure of C1H and the bottomhole flowing pressure of C2H.Since the reserves on the left side of Sandbody C1 are not affected by any wells other than C1H and C2H, the pressure variation between the two wells is mainly controlled by injection from C1H and production from C2H.The pressure can be regarded as decreasing uniformly from Well C1H to Well C2H. Accordingly, the pressure profile of the ten streamtubes is assigned with a uniform linear decline.

Third, using the model parameters determined in the above two steps, a fully implicit finite-difference numerical solution is adopted to calculate the moving profiles of gas saturation in the ten streamtubes. Figure 10 shows the simulated fluid saturation variation profiles of the ten streamtubes between Wells C1H and C2H.

Based on calculations over 100 time steps, the fluid saturation migration profiles of the ten streamtubes at time steps of 20, 40, 60 and 80 are plotted respectively, as shown in Fig. 10 Due to the large permeability contrast in the reservoir, the advancement speeds of the gas displacement front in the ten streamtubes also differ significantly.

**Fig. 11:**
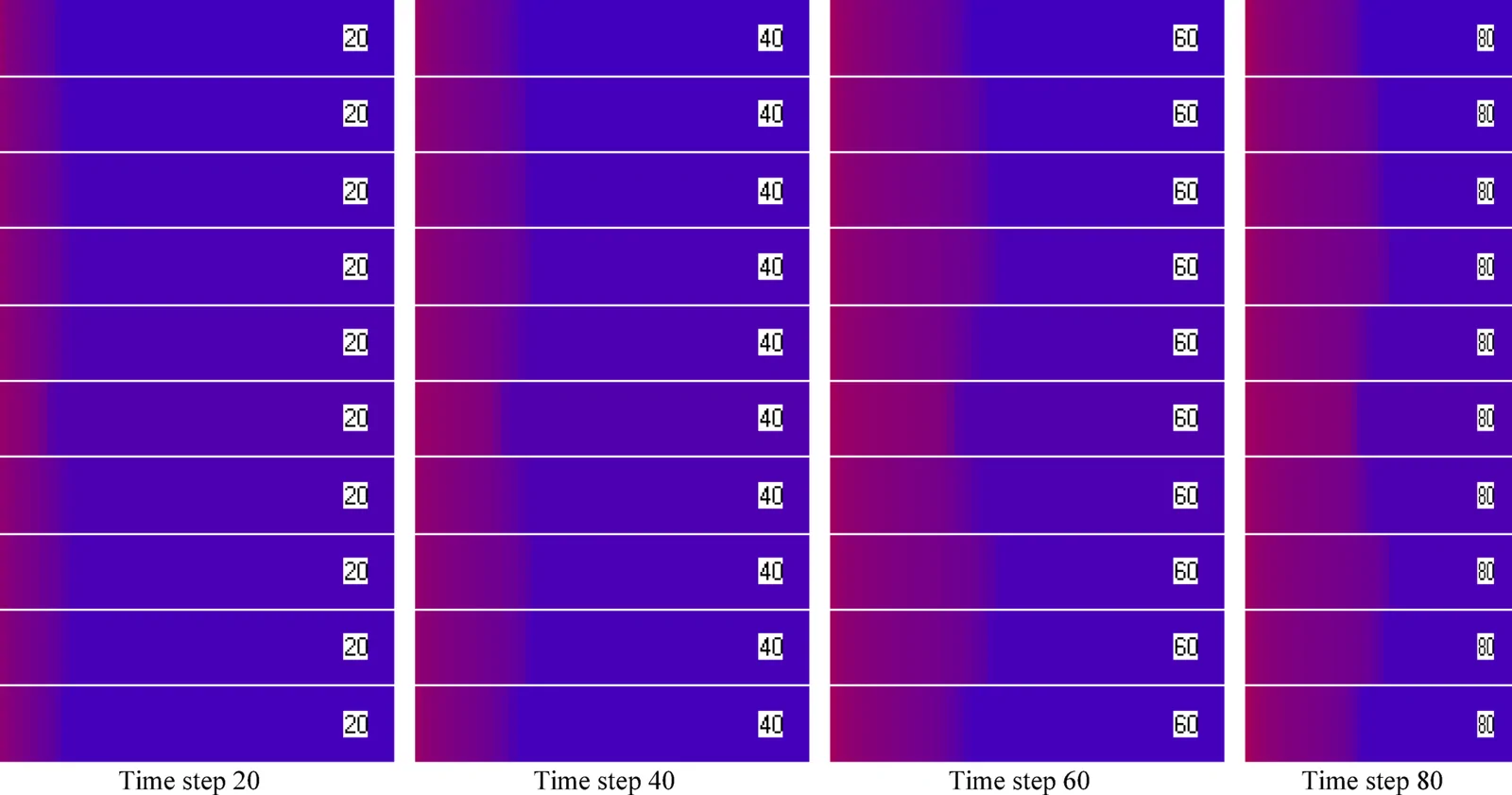
Fluid saturation profile of Diagrams of gas flooding at different time steps 20, 40, 60 and 80.

An in-depth analysis was performed on the cumulative production and original volume of the ten stream tubes. The relationship curve between recovery ratio and gas-oil ratio (GOR) was calculated and compared with the actual curves of recovery factor and GOR variation for the C1H-C2H well group, as shown in Fig. 11. The theoretical calculations are in good agreement with the field data, with a root mean square error (RMSE) of less than 3%, which demonstrates the rationality of the establishment and calculation of the theoretical model. Meanwhile, it is recognized that for immiscible gas flooding, the gas breakthrough velocity and breakthrough time are key development indicators that require special attention in reservoir development. Furthermore, based on the calculations of the above theoretical model, we weakened the heterogeneity of the ten stream tubes by removing the influence of continuous high-permeability streaks and then conducted fully implicit theoretical calculations. However, it was consistently impossible to obtain GOR variation data close to the actual field data. This indicates that when establishing a multi-stream tube model, it is essential to extract the actual key reservoir characteristic information and incorporate it into the stream tube model parameters to achieve satisfactory fitting or prediction results.

## Conclusion

(1) A novel fully implicit numerical solution method for the Buckley-Leverett equation is proposed, and the detailed procedures of the theoretical calculation approach are elaborated.

(2) A multi-streamtube model is established and solved with the fully implicit numerical method, which can equivalently simulate the influence of reservoir heterogeneity on displacement.

(3) Considering the effect of the gas equation of state, volume conservation is converted into mass conservation, on the basis of which the Buckley-Leverett equation can be applied to calculate the fluid saturation distribution during immiscible gas flooding.

(4) Combined with actual case analysis, the method for calculating immiscible gas flooding fluid saturation distribution based on full implicit computation is practically feasible and can provide reference and guidance for similar relevant studies.

## Data Availability

The datasets used and/or analyzed during the current study available from the corresponding author on reasonable request.
